# Supplementary material for: Effective combination of arugula vermicompost, chitin and inhibitory bacteria for suppression of the root-knot nematode Meloidogyne javanica and explanation of their beneficial properties based on microbial analysis
Source: PLoS One. 2023 Aug 16;18(8):e0289935. doi: 10.1371/journal.pone.0289935 (PMC10431669; doi:10.1371/journal.pone.0289935)
Supplement: S1 Fig — (DOCX) [file pone.0289935.s001.docx]

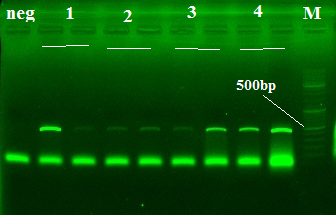


**First round**


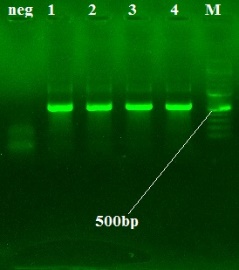


**Second round**

S1 Fig- Electrophoresis pattern of V3-V4 region of 16S rDNA gene of bacteria in compost and vermicompost of arugula (Left: first round; Right: second round). M: ladder; neg: negative control (water); 1-2: samples of arugula compost; 3-4: samples of arugula vermicompost.

vermicompost.
