# Supplementary material for: Effective combination of arugula vermicompost, chitin and inhibitory bacteria for suppression of the root-knot nematode Meloidogyne javanica and explanation of their beneficial properties based on microbial analysis
Source: PLoS One. 2023 Aug 16;18(8):e0289935. doi: 10.1371/journal.pone.0289935 (PMC10431669; doi:10.1371/journal.pone.0289935)
Supplement: S2 Fig — Euclidean distance as a similarity metric and complete linkage method. (M: Arugula compost; VM: Arugula vermicompost). (DOCX) [file pone.0289935.s002.docx]

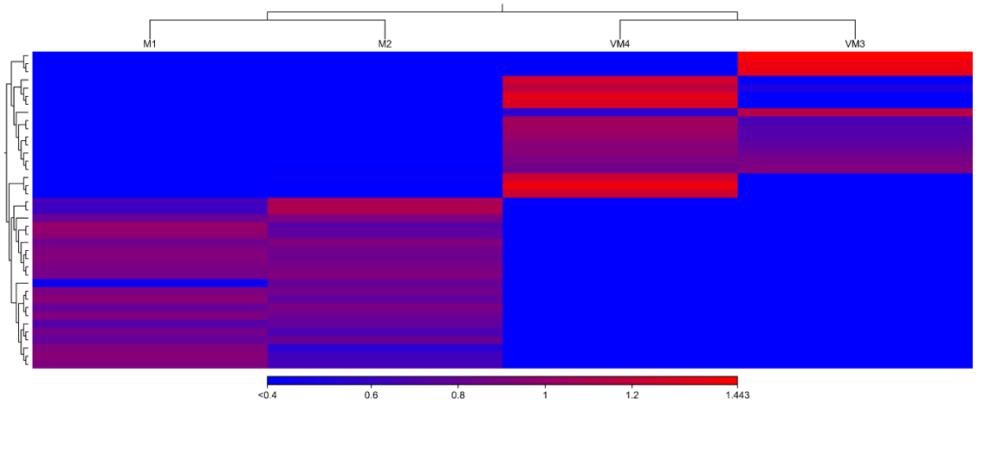


S2 Fig- The different abundance of bacterial community differential heat map of vermicompost and compost of arugula. Euclidean distance as similarity metric and complete linkage method. M: arugula compost; VM: arugula vermicompost.
