# Supplementary material for: Effective combination of arugula vermicompost, chitin and inhibitory bacteria for suppression of the root-knot nematode Meloidogyne javanica and explanation of their beneficial properties based on microbial analysis
Source: PLoS One. 2023 Aug 16;18(8):e0289935. doi: 10.1371/journal.pone.0289935 (PMC10431669; doi:10.1371/journal.pone.0289935)
Supplement: S1 Table — (DOCX) [file pone.0289935.s003.docx]

S1 Table- Sequences of the primer sets used for amplifying V3-V4 region of 16S rDNA gene of bacteria.

|  | Primer name | Sequences |
| --- | --- | --- |
| **First round primers** | 515f | TCG TCG GCA GCG TCA GAT GTG TAT AAG AGA CAG GTG YCA GCM GCC GCG GTA A |
|  | 926R | GTC TCG TGG GCT CGG AGA TGT GTA TAA GAG ACA GCC GYC AAT TYM TTT RAG TTT |
| **Second round primers** | S 502 (forward) | AAT GAT ACG GCG ACC ACC GAG ATC TAC ACC TCT CTA TTC GTC GGC AGC GTC |
|  | N 701 (revers) | CAA GCA GAA GAC GGC ATA CGA GAT TCG CCT TAG TCT CGT GGG CTC GG |
|  | N 702 (revers) | CAA GCA GAA GAC GGC ATA CGA GAT CTA GTA CGG TCT CGT GGG CTC GG |
|  | N 703 (revers) | CAA GCA GAA GAC GGC ATA CGA GAT TTC TGC CTG TCT CGT GGG CTC GG |
|  | N 704 (revers) | CAA GCA GAA GAC GGC ATA CGA GAT GCT CAG GAG TCT CGT GGG CTC GG |
