# Supplementary material for: Effective combination of arugula vermicompost, chitin and inhibitory bacteria for suppression of the root-knot nematode Meloidogyne javanica and explanation of their beneficial properties based on microbial analysis
Source: PLoS One. 2023 Aug 16;18(8):e0289935. doi: 10.1371/journal.pone.0289935 (PMC10431669; doi:10.1371/journal.pone.0289935)
Supplement: S2 Table — (DOCX) [file pone.0289935.s004.docx]

S2 Table- Thermal program for amplifying V3-V4 region of 16S rDNA gene of bacteria.

|  | Temperature (°C) | | Number of cycles | |  |
| --- | --- | --- | --- | --- | --- |
| Stage | First round | Second round | First-round | Second round | Time |
| Initial denaturation | 95 | 95 | 1 | 1 | 5 min |
| Denaturation | 95 | 95 | 30 | 15 | 30 s |
| Annealing | 50 | 55 | 30 | 15 | 30 s |
| Extension | 72 | 72 | 30 | 15 | 40 s |
| Final extension | 72 | 72 | 1 | 1 | 5 min |
