# Supplementary figures and images for: Effective combination of arugula vermicompost, chitin and inhibitory bacteria for suppression of the root-knot nematode Meloidogyne javanica and explanation of their beneficial properties based on microbial analysis
Source: PLoS One. 2023 Aug 16;18(8):e0289935. doi: 10.1371/journal.pone.0289935 (PMC10431669; doi:10.1371/journal.pone.0289935)

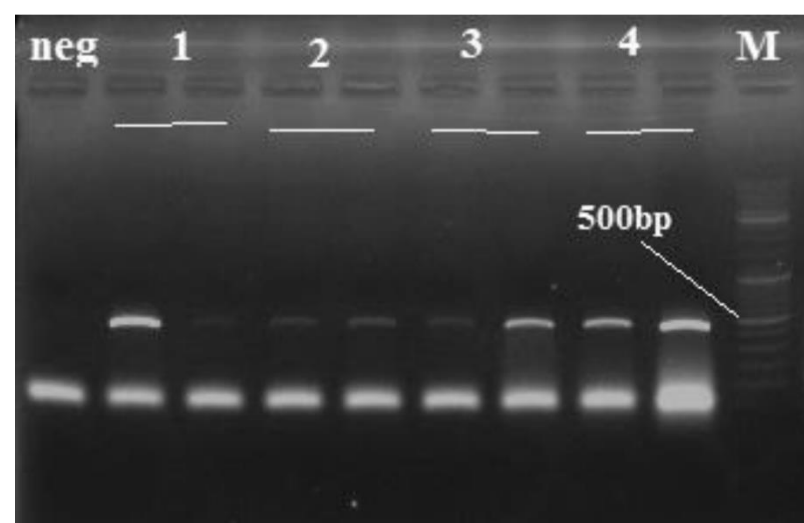

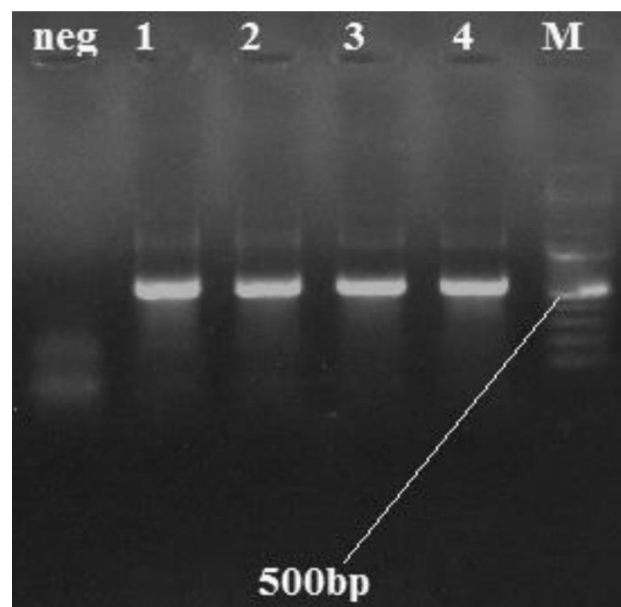

Supplement: S1 Raw images — (PDF) [file pone.0289935.s005.pdf]
