# Supplementary material for: Effective combination of arugula vermicompost, chitin and inhibitory bacteria for suppression of the root-knot nematode Meloidogyne javanica and explanation of their beneficial properties based on microbial analysis
Source: PLoS One. 2023 Aug 16;18(8):e0289935. doi: 10.1371/journal.pone.0289935 (PMC10431669; doi:10.1371/journal.pone.0289935)
Supplement: Attachment — Submitted filename: Review_comments.docx [file pone.0289935.s007.docx]

Major Comments:

The introduction should be restructured to clearly outline the purpose of the research and its significance. It should highlight the problem of nematode infestation in agriculture and the need for eco-friendly and sustainable solutions.

A comprehensive literature review should be conducted to explore existing research on the use of vermicompost as a biocontrol agent against nematodes. Cite relevant studies that have demonstrated the efficacy of vermicompost in nematode control. Discuss the limitations and gaps in the current knowledge that this research aims to address.

Minor Comments:

1) "The earthworms could not feed on the castor bean plant, and its vermicompost was not produced." - Justification for this statement should be included, explaining why earthworms couldn't feed on the castor bean plant and how it impacted vermicompost production.
2) "The predominant bacterial phylum in vermicompost belongs to a gram-negative group" - Mention the specific name of the gram-negative group.
3) "This study was conducted with to introduce an effective combination to control the damage caused by the root-knot nematode." - Correct the sentence for clarity, possibly: "This study aims to introduce an effective combination for controlling root-knot nematode damage."
4) English language and writing should be improved, possibly by seeking the assistance of a native speaker for proofreading and editing. This will help ensure clarity and proper communication of the research findings.
