# Supplementary material for: Effective combination of arugula vermicompost, chitin and inhibitory bacteria for suppression of the root-knot nematode Meloidogyne javanica and explanation of their beneficial properties based on microbial analysis
Source: PLoS One. 2023 Aug 16;18(8):e0289935. doi: 10.1371/journal.pone.0289935 (PMC10431669; doi:10.1371/journal.pone.0289935)
Supplement: Attachment — Submitted filename: Response to Reviewers-2.docx [file pone.0289935.s008.docx]

July 26, 2023

Prof. Raja Aadil Hussain Bhat

Editor in Chief

PLOS ONE

Dear Prof. Raja Aadil Hussain Bhat,

First, my co-authors and I would like to thank you and the two anonymous reviewers for considering our manuscript PONE-D-22-34501 with a positive attitude. We are very grateful to the reviewers for their comments, which clearly helped us to improve the quality of this manuscript. We agree with the reviewers on many points and have listed our response point by point at the end of this letter to explain why.

We hope that both you and the reviewers appreciate our revised text, and we are always available if you need further clarification on the revised text. For your information, all changes in the text are marked as "Highlight Text." Below are the reviewers' comments, and our responses (RED text). Also, we check the adjustment of figures manuscript by the Preflight Analysis and Conversion Engine (PACE) that figures met PLOS requirements. We reviewed references and they also were checked by "http://retractiondatabase.org".

We thank you again for your time and attention and look forward to making a final decision on the manuscript.

Kind regards

Major Comments:

The introduction should be restructured to clearly outline the purpose of the research and its significance. It should highlight the problem of nematode infestation in agriculture and the need for eco-friendly and sustainable solutions.

A comprehensive literature review should be conducted to explore existing research on the use of vermicompost as a biocontrol agent against nematodes. Cite relevant studies that have demonstrated the efficacy of vermicompost in nematode control. Discuss the limitations and gaps in the current knowledge that this research aims to address.

In response to your suggestion, we have restructured the introduction to more explicitly outline the purpose of our research and emphasize its significance in addressing the problem of nematode infestation in agriculture. The revised introduction now provides a more comprehensive overview of the importance of eco-friendly and sustainable solutions for managing nematode pests and the utilization of vermicompost as a biocontrol agent based on existing research findings. The relevant references were added to the manuscript.

2: Pajovic, I., Širca, S., Stare, B. G., & Urek, G. (2007). The incidence of root-knot nematodes Meloidogyne arenaria, M. incognita, and M. javanica on vegetables and weeds in Montenegro. Plant disease, 91(11), 1514-1514.

3: Pathak, V. M., Verma, V. K., Rawat, B. S., Kaur, B., Babu, N., Sharma, A., ... & Cunill, J. M. (2022). Current status of pesticide effects on environment, human health and it’s eco-friendly management as bioremediation: A comprehensive review. Frontiers in Microbiology, 2833.

4: Forghani, F., & Hajihassani, A. (2020). Recent advances in the development of environmentally benign treatments to control root-knot nematodes. Frontiers in plant science, 11, 1125.

8: Mondal, S., Ghosh, S., & Mukherjee, A. (2021). Application of biochar and vermicompost against the rice root-knot nematode (Meloidogyne graminicola): an eco-friendly approach in nematode management. Journal of Plant Diseases and Protection, 128, 819-829.

9: Liu, D., Han, W., Zhang, Y., & Jiang, Y. (2019). Evaluation of vermicompost and extracts on tomato root-knot nematode. Bangladesh Journal of Botany, 48(3 Special), 845-851.

10: Zuhair, R., Moustafa, Y. T. A., Mustafa, N. S., El-Dahshouri, M. F., Zhang, L., & Ageba, M. F. (2022). Efficacy of amended vermicompost for bio-control of root knot nematode (RKN) Meloidogyne incognita infesting tomato in Egypt. Environmental Technology & Innovation, 27, 102397.

11: Safiddine, F., Nebih, D., Merah, O., & Djazouli, Z. E. (2019). The impact of different vermicompost types on reducing the number of Meloidogyne root-knot nematodes and the vegetative expression of tomato plants. AgroBiologia, 9(1), 1415-1427.

Minor Comments:

1) "The earthworms could not feed on the castor bean plant, and its vermicompost was not produced." - Justification for this statement should be included, explaining why earthworms couldn't feed on the castor bean plant and how it impacted vermicompost production.

This was done and the relevant reference was added to the manuscript.

The fresh leaves of the plant cannot be utilized as earthworm feed due to their tendency to become soggy, decay, and release harmful exudates that are lethal to earthworms.

37. Antony Godson SG, Gajalakshmi S. exploring the possibility of utilizing castor bean (*Ricinus communis*) plant leaves in vermicomposting with three epigeic earthworm species. J Emerg Technol Innov Res.2018; 12(5): 501-507.

38. Mahadev ND, Thorat AT, Vitthal BP. An evaluation of anthelmintic activity of *Ricinus communis* Linn. leaves by using different type of solvent. J Pharmacogn Phytochem. 2017;6(4):1845-7.

2) "The predominant bacterial phylum in vermicompost belongs to a gram-negative group" - Mention the specific name of the gram-negative group.

This was done

The predominant bacterial phylum in vermicompost belongs to *Proteobacteria*

3) "This study was conducted with to introduce an effective combination to control the damage caused by the root-knot nematode." - Correct the sentence for clarity, possibly: "This study aims to introduce an effective combination for controlling root-knot nematode damage."

It was corrected in the text.

4) English language and writing should be improved, possibly by seeking the assistance of a native speaker for proofreading and editing. This will help ensure clarity and proper communication of the research findings.

All authors have revised the manuscript to check its spelling and English structure. We have improved the text as much as possible. The changes are highlighted in the text.
